# Exercise for Bone Mineral Density in People with Inflammatory Bowel Disease: A Systematic Review

**DOI:** 10.3390/healthcare14111448

**Published:** 2026-05-24

**Authors:** Joaquín González-Aroca, Jorge Olivares-Arancibia, Rodrigo Quera, Walter Sepúlveda-Loyola, Cristian Barros-Osorio, Júlio Brugnara Mello, José Francisco López-Gil, Julio Plaza-Diaz

**Affiliations:** 1 Escuela de Kinesiología, Facultad de Salud, Universidad Santo Tomás, La Serena 1720236, Chile; jgonzalez180@santotomas.cl; 2 Faculty of Sciences, Universidad de La Serena, La Serena 1720236, Chile; 3 AFySE Group (Physical Activity, Physical Fitness and School Health), Research in Physical Activity and School Health, School of Physical Education, Faculty of Education, Universidad de las Américas, Santiago 7500975, Chile; jorge.olivares.ar@gmail.com; 4 Inflammatory Bowel Disease Program, Clínica Universidad de Los Andes, Santiago 7500975, Chile; rquera@uandes.cl; 5 Faculty of Health and Social Sciences, Center for Research in Biological and Chemical Sciences, Universidad de Las Américas, Santiago 7500975, Chile; wsepulveda@udla.cl (W.S.-L.); neurociencias.cristianbarros@gmail.com (C.B.-O.); 6 Grupo de Investigación en Adaptaciones Musculoesqueléticas al Entrenamiento, Escuela de Educación Física, Pontificia Universidad Católica de Valparaíso, Valparaíso 2340025, Chile; julio.mello@pucv.cl; 7 School of Medicine, Universidad Espíritu Santo, Samborondón 092301, Ecuador; 8 Vicerrectoría de Investigación y Postgrado, Universidad de Los Lagos, Osorno 5290000, Chile; 9 School of Health Sciences, Universidad Internacional de La Rioja, 26006 Logroño, Spain; julioramon.plaza@urv.cat; 10 Departament de Bioquímica i Biotecnologia, ANUT-DSM (Alimentació, Nutrició Desenvolupament i Salut Mental), Universitat Rovira i Virgili, 43201 Reus, Spain; 11 Institut de Recerca Biomèdica Catalunya Sud, Hospital Universitari Sant Joan de Reus, 43204 Reus, Spain; 12 Biomedical Research Networking Center for Physiopathology of Obesity and Nutrition (CIBERObn), Institute of Health Carlos III, 28029 Madrid, Spain

**Keywords:** inflammatory bowel disease, areal bone mineral density, exercise, systematic review

## Abstract

Background/Objectives: Inflammatory bowel disease (IBD) is associated with reduced areal bone mineral density (aBMD) and an increased risk of osteoporosis and fragility fractures. Although exercise improves bone health in the general population, its effects on aBMD in adults with IBD are unclear. This systematic review aimed to evaluate the effects of structured exercise interventions on aBMD in adults with IBD and to assess the certainty of the evidence. Methods: We conducted a systematic review in accordance with Preferred Reporting Items for Systematic Reviews and Meta-Analyses (PRISMA) guidelines and the Cochrane Handbook. Searches were performed in CENTRAL, MEDLINE, Scopus, and Web of Science from inception to November 2025. We included randomized controlled trials comparing structured exercise interventions with usual care, no structured exercise or no intervention in participants aged 16 years and older with IBD. The primary outcome was aBMD; physical activity was a secondary outcome. Risk of bias was assessed using the Cochrane Risk of Bias tool (RoB 2.0), and certainty of evidence was evaluated using Grading of Recommendations Assessment, Development and Evaluation (GRADE). The review protocol was registered in International Prospective Register of Systematic Reviews (PROSPERO) CRD42024617200. Results: Two randomized controlled trials (*n* = 164), both conducted exclusively in adults with Crohn’s disease, met the inclusion criteria. Combined impact and resistance training for 6 months was associated with greater lumbar spine aBMD compared with usual care, while hip outcomes were not consistently improved. A 12-month low-impact exercise program compared with no intervention suggested greater trochanter aBMD gain among fully compliant participants, but intention-to-treat between-group differences were not statistically significant across skeletal sites. Due to heterogeneity in interventions and reporting, meta-analysis was not performed. Overall certainty of the evidence was very low because of methodological limitations and imprecision. Conclusions: We are very uncertain about the effect of exercise interventions on aBMD in adults with IBD. Current randomized evidence is limited to adults with Crohn’s disease and is insufficient to determine the optimal exercise modality, frequency, intensity, progression, or loading characteristics for improving bone health. Well-designed trials across IBD phenotypes are needed to clarify the role of exercise in bone health management in IBD.

## 1. Introduction

Inflammatory bowel disease (IBD) refers to a group of chronic, relapsing inflammatory disorders affecting the gastrointestinal tract, primarily including ulcerative colitis (UC), Crohn’s disease (CD), and unclassified IBD. The prevalence of IBD surpasses 0.3% in Europe, North America, and Oceania, with its incidence increasing rapidly in newly industrialized countries [1]. CD and UC are the most prevalent forms of IBD. Both conditions exhibit cycles of relapse and remission and share some pathological and clinical characteristics while also presenting distinct differences [2]. People with CD or UC commonly experience symptoms such as diarrhea, abdominal pain, fatigue, weight loss, and rectal bleeding [3].

There is evidence linking IBD to reduced areal bone mineral density (aBMD) and an increased risk of osteoporosis. Observational studies have reported decreased aBMD in individuals with IBD, along with a higher prevalence of osteoporosis as the disease advances [4]. Bone loss in IBD is multifactorial and has been attributed to systemic inflammation, malnutrition and low body mass, micronutrient deficiencies (particularly calcium and vitamin D), hypogonadism, and cumulative exposure to glucocorticoids and/or intestinal resection [5]. Additionally, treatments like corticosteroid therapy and prior surgery further increase patients’ susceptibility to osteoporosis [6].

Adults with IBD have significantly lower aBMD and a higher risk of fragility fractures compared with healthy controls, particularly vertebral fractures [7]. Recent meta-analytic data indicate that approximately 12% of adults with IBD present with osteoporosis and more than 30% with osteopenia, underscoring the magnitude of skeletal involvement in this population [8]. Consistent with this burden, clinical guidance recommends bone-health risk assessment and lifestyle measures, including weight-bearing exercise (along with smoking cessation and adequate calcium/vitamin D intake), to help prevent bone loss in IBD [9].

Despite this burden, most available evidence has focused on prevalence and fracture risk, while the role of exercise as a therapeutic strategy to improve bone health in IBD remains insufficiently explored.

Although reduced aBMD and fracture risk in IBD have been extensively described, the effects of exercise interventions on aBMD remain insufficiently evaluated. Existing systematic reviews have mainly summarized intervention studies without formally assessing the certainty of the evidence [10,11]. As a result, clinicians lack clear guidance regarding the strength of the evidence supporting exercise-based recommendations for bone health in IBD. To address this limitation, the present systematic review aimed not only to evaluate the effects of exercise interventions on aBMD in adults with IBD, but also to appraise the certainty of the evidence for this outcome using the Grading of Recommendations Assessment, Development and Evaluation (GRADE) approach.

## 2. Materials and Methods

This systematic review was conducted following the guidelines of the Preferred Reporting Items for Systematic Reviews and Meta-Analyses (PRISMA) [12] and the Cochrane handbook for systematic reviews of interventions [13]. The review protocol was registered in International Prospective Register of Systematic Reviews (PROSPERO) CRD42024617200.

Population, Intervention, Comparator, Outcomes, Study design (PICOS) framework:

P (Population): People (≥16 years) with IBD (UC, CD, or IBD-unclassified), as defined by study authors.

I (Intervention): Structured exercise programs (planned, structured, repetitive bodily movement intended to improve/maintain fitness).

C (Comparator): Usual care/no structured exercise or no intervention.

O (Outcomes): Primary—aBMD (ideally measured by dual-energy X-ray absorptiometry [DXA] at lumbar spine/hip); secondary—physical activity (as reported by included trials).

S (Study design): Randomized controlled trials (RCTs) (parallel-group, cross-over, or cluster-randomized).

### 2.1. Criteria for Considering Studies for This Review

#### 2.1.1. Types of Studies

We included all published and unpublished RCTs with parallel or cross-over design that compared exercise interventions targeted at people with IBD to usual care, no structured exercise, other non-exercise comparators, or no intervention. Eligible studies included both individual and cluster randomized designs. We excluded observational studies.

#### 2.1.2. Types of Participants

We included people (≥16 years) with IBD (as defined by the study authors).

#### 2.1.3. Types of Intervention

We included any type of exercise program as defined by the American College of Sports Medicine (ACSM) 2014 as “planned, structured, repetitive bodily movement performed to improve or maintain one or more components of physical fitness” [14].

#### 2.1.4. Types of Outcomes

Our primary outcome was aBMD, and our secondary outcome was physical activity. Where available, we extracted aBMD by skeletal site (lumbar spine, femoral neck, total hip/greater trochanter) and the assessment method (e.g., DXA).

### 2.2. Search Methods for Identification of Studies

We searched the following sources from the inception of each database to 30 November 2025:

- CENTRAL;
- MEDLINE (PubMed);
- Scopus;
- Web of Science.

We placed no restrictions on language of publication. For detailed search strategies, see Appendix A. As complementary search methods, we checked relevant systematic reviews for potentially eligible studies. We also scrutinized the references of included studies.

### 2.3. Data Collection and Analysis

We carried out data collection and analysis according to the methods recommended in the Cochrane Handbook for Systematic Reviews of Interventions.

#### 2.3.1. Selection of Studies

Two authors independently (JG and JO) screened the titles and abstracts of all studies identified in the updated electronic search using Rayyan web application [15] (Rayyan Systems Inc., Cambridge, MA, USA). Available from: https://www.rayyan.ai/, accessed on 20 June 2025. Full reports were obtained for studies that appeared to meet the inclusion criteria or for which the title and abstract lacked sufficient information to make a clear decision. The two authors then independently assessed these full reports to determine if the studies met the inclusion criteria. Any disagreements were resolved through discussion with a third reviewer (RQ).

#### 2.3.2. Data Extraction and Management

Two review authors independently carried out data extraction using piloted data extraction forms. We extracted relevant data from full-text articles that met the inclusion criteria including:

- Trial setting: Country and number of trial center;
- Methods: Study design, objectives;
- Participant characteristics: Age, socio-demographics, ethnicity, diagnostic criteria; disease activity;
- Eligibility criteria: Inclusion and exclusion criteria;
- Intervention and comparator;
- Patient outcomes;
- Results: Number of participants allocated to each group, missing participants, sample size.

### 2.4. Assessment of Risk of Bias in Included Studies

Two review authors independently assessed the risk of bias using version 2 of the Cochrane risk of bias tool for RCTs (RoB 2) [16]. This tool assesses bias across five domains: bias arising from the randomization process; bias due to deviations from intended interventions; bias due to missing outcome data; bias in measurement of the outcome; and bias in selection of the reported result.

We assessed each domain as having low risk of bias, high risk of bias, or some concerns. We contacted study authors if insufficient information was provided to permit the risk of bias assessment. If study authors were unable to provide data, and the information was insufficient to assess the risk of bias for a specific domain, we described that domain as having some concerns.

Because participant and intervention-provider blinding is generally not feasible in structured exercise trials, lack of participant blinding was not considered sufficient by itself to judge an objective DXA-derived aBMD outcome as having high risk of bias. We therefore interpreted RoB 2.0 domains according to outcome type. For the primary outcome, aBMD measured by DXA was considered less susceptible to measurement bias than self-reported outcomes such as physical activity, fatigue, or quality of life. When relevant, we distinguished risk-of-bias concerns for objective aBMD outcomes from those for participant-reported secondary outcomes.

### 2.5. Measures of Treatment Effect

We had planned to conduct a meta-analysis of the collected data. However, this was not possible because the identified outcome measures were not comparable in terms of time and comparison. For interpretability in the preparation of the summary of findings table, the between-group mean differences (MDs) in aBMD reported in absolute units (g/cm^2^) were additionally expressed as percentage differences. This conversion was performed by dividing the adjusted between-group MD by the baseline aBMD value of the control group at the corresponding skeletal site and multiplying the result by 100. The same approach was applied to the lower and upper limits of the 95% confidence intervals (CIs).

These percentage values were used for interpretability only and were not used to change statistical inferences. The primary effect estimates remained the adjusted between-group MDs in aBMD reported in absolute units (g/cm^2^), when available.

### 2.6. Investigation of Heterogeneity and Subgroup Analysis

We were not able to investigate heterogeneity or perform planned subgroup analyses because only two trials were included.

### 2.7. Certainty of the Evidence Assessment

We constructed two summaries of findings tables, using the GRADE [17] approach to provide recommendations incorporating relevance, applicability, and certainty of evidence from this review [18]. For our primary outcome (aBMD), we reported the percentage of change (with 95% CI). Also, we presented the number of trials contributing to the outcome and the number of participants. Furthermore, the GRADE approach was used to assess the certainty of the body of evidence for each outcome in the summary of findings tables.

We assessed the certainty of evidence based on: the risk of bias within the studies contributing to the outcome (using our RoB 2.0 assessments); the relevance to our population of interest (indirectness); unexplained heterogeneity or inconsistency; imprecision of the results; and high risk of publication bias. In GRADE, a body of evidence from RCTs begins with a high-certainty rating.

We then downgraded the evidence by one level if we deemed the risk of bias to be serious, and by two levels if we deemed the risk to be very serious.

We assessed the outcome individually and gave it a high, moderate, low or very low rating for the certainty of evidence, using the following definitions.

1. High certainty: We are very confident that the true effect lies close to that of the estimate of the effect.
2. Moderate certainty: We are moderately confident in the effect estimate: the true effect is likely to be close to the estimate of the effect, but there is a possibility that it is substantially different.
3. Low certainty: Our confidence in the effect estimate is limited: the true effect may be substantially different from the estimate of the effect.
4. Very low certainty: We have very little confidence in the effect estimate: the true effect is likely to be substantially different from the estimate of effect. Where we downgraded the certainty of the evidence, we described our reasons and the supporting information for our decisions in the footnotes of the summary of findings tables. If it had been necessary, we would have contacted the study authors for clarification of details to allow judgements to be made.

## 3. Results

### 3.1. Results of the Search

**Figure 1:**
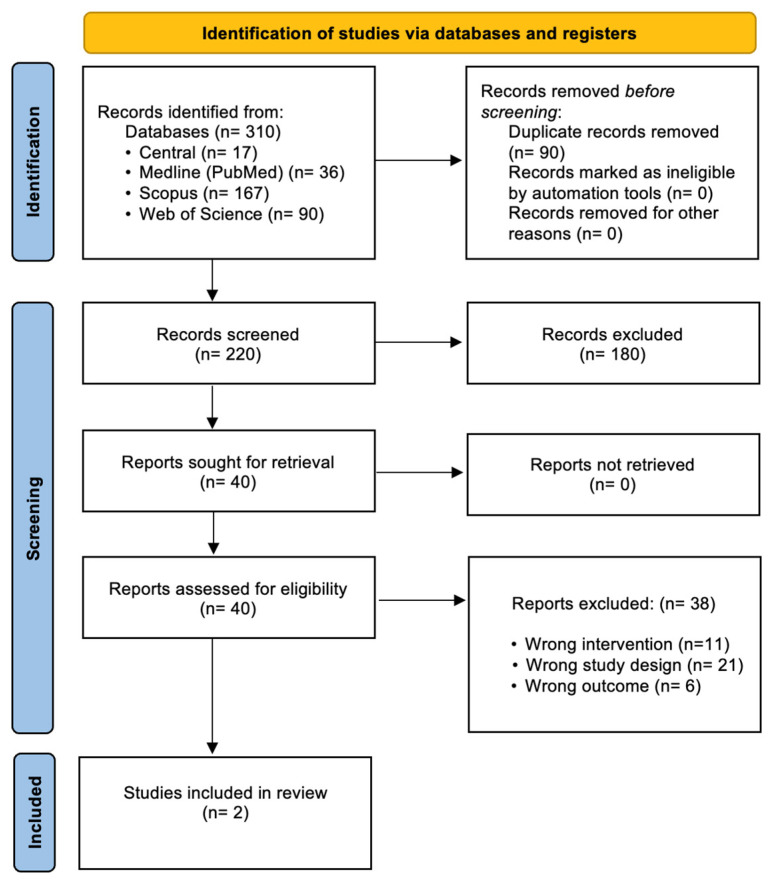
Flow diagram (PRISMA) of the selection process.

We completed our literature search in November 2025, identifying a total of 310 records. After removal of duplicates, 220 records remained. Title and abstract screening identified 40 records for full-text review (180 records were excluded at this stage). After assessing all 40 records, we identified two studies that met the inclusion criteria and were included in the review. We excluded 38 records (wrong outcome, n = 6; wrong intervention, *n* = 11; wrong study design, *n* = 21). The results of the search are presented in a PRISMA flow diagram (Figure 1).

### 3.2. Included Studies

#### 3.2.1. Setting

Two RCTs involving a total of 164 participants (61.5% females) met our inclusion criteria. Both studies were conducted in England. One study [19] was conducted in a university exercise science laboratory, and the other study [20] was conducted at home; however, this study did not provide information on where outcomes were measured (Table 1).

#### 3.2.2. Participants

Age ranged from 16 years [19] to 65 years old [20]. The two included studies examined exclusively CD populations. One study examined participants in both active and inactive states of the disease [19] and the other study examined participants in an inactive state of the disease [20]. One study [19] reported the disease activity of its participants as a mean value using the Crohn’s Disease Activity Index (CDAI) and Robinson et al. [20] reported a mean using the Harvey–Bradshaw Index (HBI) for CD. Only Jones et al. [19] had trial registration (ISRCTN11470370).

#### 3.2.3. Interventions

Both studies implemented exercise programs aimed at enhancing bone health by targeting key muscle groups and loading the hip and lumbar spine. Robinson et al. [20] used a home-based, low-impact program with 12 floor-based exercises focusing on dynamic conditioning of the trunk and leg muscles, including the quadriceps, hamstrings, gluteals, and erector spinae. Participants were encouraged to exercise at least twice a week, with intensity progressively increased through additional repetitions, advanced body positions, and the use of resistance tools like bands or weights. Support meetings were held at 1, 3, and 9 months to maintain motivation and adjust training.

Jones et al. [19] combined supervised and unsupervised sessions over 26 weeks, tapering supervision to promote self-management in a home-based program. Participants completed three 60 min sessions weekly, starting with more frequent supervision and shifting to one monthly session from week 9 onward. Each session included a warm-up, a main conditioning phase, and a cool-down. The main phase integrated impact activities (e.g., jumping and rope skipping) and resistance exercises targeting major muscle groups, with intensity monitored using the Resistance Intensity Scale. Resistance was provided through body weight and elastic bands, aiming for moderate-to-hard effort throughout the program.

#### 3.2.4. Outcomes

##### Areal Bone Mineral Density

Both studies assessed aBMD using DXA at the hip and lumbar spine, with measurements taken at baseline and follow-up (6 months in the first study and 12 months in the second). Jones et al. [19] measured aBMD at the femoral neck, greater trochanter, and lumbar spine, reporting excellent reliability (intraclass correlation coefficient [ICC] > 0.998). Robinson et al. [20] also measured aBMD at Ward’s triangle, and all measurements were performed by a single operator, with coefficients of variation below 2.2%.

##### Physical Activity

Physical activity was assessed as a secondary outcome in both included RCTs. In Jones et al. [19], habitual physical activity was assessed using self-reported measures of time spent in moderate-to-vigorous physical activity at baseline and after six months. In contrast, Robinson et al. [20] evaluated habitual physical activity using a structured interviewer-administered questionnaire based on the Allied Dunbar National Fitness Survey. Participants were categorized into activity levels according to weekly energy expenditure expressed in metabolic equivalent units, with assessments conducted at baseline and after 12 months.

##### Adverse Events

Jones et al. [19] reported three exercise-related adverse events.

### 3.3. Risk of Bias in Included Studies

**Figure 2:**
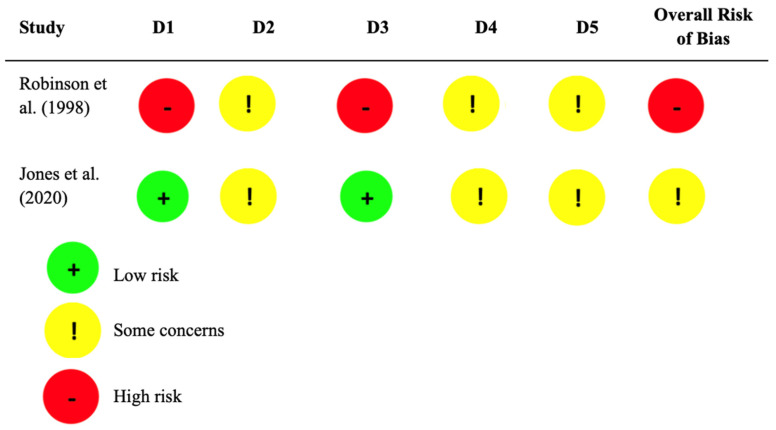
The Cochrane risk-of-bias tool for randomized trials (RoB 2.0). Abbreviations: D1, randomization process; D2, deviations from intended interventions; D3, missing outcome data; D4, outcome measurement; D5, selection of the reported result [19,20].

Below we present the results of our risk of bias assessment (Figure 2). Further details about the judgment of each domain can be found in the risk of bias tables (Appendix B).

### 3.4. Effect of Interventions

#### 3.4.1. Exercise Compared with Usual Care

See Table 2 for summary of findings.

##### Areal Bone Mineral Density

One RCT [19] evaluated the effects of a six-month combined impact and resistance training program on aBMD in adults with stable CD. At six months, participants allocated to the exercise intervention demonstrated significantly greater lumbar spine aBMD than those in the control group receiving usual care (adjusted MD 0.036 g/cm^2^, 95% CI 0.024 to 0.048; *p* < 0.001). In contrast, no statistically significant between-group differences were detected at the femoral neck (adjusted MD 0.018 g/cm^2^, 95% CI 0.001 to 0.035; *p* = 0.059) or greater trochanter (adjusted MD 0.013 g/cm^2^, 95% CI −0.019 to 0.045; *p* = 0.415) after correction for multiple comparisons.

##### Physical Activity

At six months, there were no statistically significant differences between the exercise and control groups in total minutes of physical activity per week (adjusted MD −21 min/week, 95% CI −499 to 457; *p* = 0.930). Similar findings were observed at three months, indicating that the structured exercise intervention did not result in sustained between-group differences in self-reported habitual physical activity outside the prescribed training sessions.

#### 3.4.2. Exercise Compared with No Intervention

See Table 3 for summary of findings.

##### Areal Bone Mineral Density

One RCT [20] evaluated the long-term effects of a 12-month home-based low-impact exercise program on aBMD in adults with CD. On an intention-to-treat basis, aBMD increased at all measured sites in the exercise group compared with controls; however, these between-group differences did not reach statistical significance at the femoral neck, lumbar spine (L2–L4), Ward’s triangle, or greater trochanter after 12 months. In a predefined subgroup analysis of participants who were fully compliant with the exercise program (≥10 sessions per month and progression of exercise intensity), greater increases in aBMD were observed across all skeletal sites compared with controls. In this compliant subgroup, the increase in aBMD at the greater trochanter was statistically significant (difference in means 4.67%, 95% CI 0.86 to 8.48; *p* = 0.02), whereas changes at the femoral neck, lumbar spine, and Ward’s triangle did not reach statistical significance. Increases in aBMD at both the hip and spine were positively associated with the number of exercise sessions completed over the 12-month period, independent of corticosteroid use, body weight, dietary intake, habitual physical activity, and disease activity.

##### Physical Activity

Habitual physical activity was assessed using a structured interview based on the Allied Dunbar National Fitness Survey to account for lifestyle activity outside the prescribed exercise program. Over the 12-month follow-up, habitual physical activity levels did not change significantly in either the exercise or control groups.

## 4. Discussion

### 4.1. Summary of Main Results

Two RCTs, including 164 participants, met the inclusion criteria for this review. Both trials were conducted exclusively in adults with Crohn’s disease, and no randomized evidence was identified in participants with ulcerative colitis or unclassified IBD. Therefore, the applicability of the findings to the wider IBD population remains uncertain. Although one trial suggested that combined impact and resistance training may improve lumbar spine aBMD compared with usual care, and another suggested possible benefits of a low-impact program among fully compliant participants, the evidence base is too limited to determine the effectiveness of exercise interventions for aBMD in IBD with confidence.

Overall, based on the available evidence, we are very uncertain about the effect of exercise interventions on aBMD in adults with IBD.

Importantly, this review should not be interpreted as identifying an optimal exercise prescription for bone health in IBD. The included interventions differed in modality, loading characteristics, supervision, duration, frequency, and progression. Because only two small trials were available, it was not possible to determine which exercise type, intensity, frequency, progression strategy, or mechanical loading profile is most appropriate for improving aBMD in this population.

Bone loss in IBD is multifactorial and cannot be attributed solely to insufficient physical activity. Chronic systemic inflammation, corticosteroid exposure, low body mass, nutritional compromise, calcium and vitamin D deficiency, hypogonadism, reduced mechanical loading, and previous intestinal resection may all contribute to impaired skeletal health. In this respect, the rationale for exercise is broadly consistent with evidence from other chronic inflammatory conditions, where resistance and impact-loading exercise may support bone health through mechanical strain and muscle-derived loading. However, IBD-specific factors, including fluctuating disease activity, gastrointestinal symptoms, malabsorption, surgery, fatigue, and corticosteroid exposure, may influence both the feasibility of exercise and the skeletal response to training. Therefore, findings from the general population or from other inflammatory diseases should be extrapolated to IBD cautiously.

Adherence may be a key determinant of skeletal benefit. In Robinson et al., greater aBMD gains were observed among participants who were fully compliant with the exercise program, and increases in aBMD were positively related to the number of exercise sessions completed. This suggests that the osteogenic effect of exercise may depend on achieving a sufficient loading dose over time rather than merely being assigned to an exercise group. At the same time, habitual physical activity did not clearly differ between intervention and control groups in the included trials. This distinction is clinically relevant because it suggests that potential skeletal benefits may arise from the specific mechanical loading characteristics of the prescribed exercise intervention, rather than from a general increase in overall physical activity.

### 4.2. Overall Completeness and Applicability of Evidence

The overall completeness and applicability of the evidence identified in this review are limited. We included only two RCTs, both conducted in adults with CD, which restricts the generalizability of the findings to other forms of IBD, such as UC or unclassified IBD. The included trials were conducted in England (a high-income country), potentially limiting their external validity to healthcare systems, lifestyle patterns, and exercise practices in other geographical regions. Differences in access to supervised exercise, cultural attitudes toward physical activity, and background levels of physical activity may influence the feasibility and effectiveness of similar interventions in other settings.

Participants in the included trials were predominantly in remission or had low disease activity, and individuals with severe or unstable disease were generally excluded.

Finally, both studies included a greater proportion of women than men. Sex-specific differences in bone metabolism, hormonal status, and fracture risk may influence the skeletal response to exercise, and these factors were not explicitly addressed in the included trials. These limitations highlight important gaps in the current evidence base and underscore the need for well-designed trials including more diverse populations, disease phenotypes, and geographical settings.

### 4.3. Quality of the Evidence

The certainty of the evidence was very low, primarily because of methodological limitations, the small number of included trials, and imprecision of the effect estimates. However, the interpretation of risk of bias requires nuance. In exercise trials, participant and intervention-provider blinding is usually infeasible and should not automatically be interpreted as high risk of bias for objective outcomes such as DXA-derived aBMD. For aBMD, risk-of-bias concerns were more closely related to randomization and allocation concealment procedures, missing outcome data, lack of protocol availability in the older trial, and possible selective reporting or incomplete analytical detail for some prespecified outcomes. By contrast, self-reported outcomes such as physical activity, fatigue, and quality of life are more susceptible to bias in unblinded exercise trials. Overall, these limitations reduce confidence in the estimated effects, and the true effect of exercise on aBMD in people with IBD may differ substantially from the estimates reported here.

### 4.4. Potential Biases in the Review Process

We conducted a comprehensive search across four major bibliographic databases. Study selection was carried out independently by two review authors, and we did not exclude otherwise eligible studies solely due to missing outcome data. Data extraction was also performed independently by two authors using a standardized extraction form, with cross-checking. The risk of bias of the included studies was assessed independently by two review authors, and any disagreements were resolved through discussion until consensus was achieved. The review was conducted in accordance with standard methods outlined in the Cochrane Handbook for Systematic Reviews of Interventions. However, clinical trial registries were not searched, and ongoing or unpublished studies may therefore not have been identified. We also did not systematically search discipline-specific databases (e.g., SPORTDiscus/CINAHL) or grey literature sources.

### 4.5. Agreements and Disagreements with Other Studies or Reviews

To date, several systematic reviews have evaluated structured exercise in IBD more broadly and have reported on DXA/aBMD outcomes when available [10,11]. Overall, our findings are broadly consistent with these previous reviews, in that both syntheses highlight the scarcity of RCTs evaluating exercise interventions and the limited evidence supporting their effects on aBMD in this population. However, the present review differs in several aspects. First, our review includes a more recent and updated search strategy, incorporating the latest available evidence. Second, whereas previous reviews provided a narrative synthesis of studies reporting aBMD outcomes, our review explicitly assessed the certainty of the evidence for aBMD using the GRADE approach.

By formally grading the certainty of the evidence, our review extends the previous syntheses by showing that the current randomized evidence remains insufficient to support firm conclusions about the direction, magnitude, or clinical relevance of exercise effects on aBMD in IBD.

## 5. Conclusions

### 5.1. Implications for Practice

We are very uncertain whether structured exercise interventions improve aBMD in people with IBD compared with usual care or no intervention, because the certainty of the evidence is very low. Current randomized evidence is limited to two trials in adults with Crohn’s disease; therefore, the applicability of these findings to ulcerative colitis, unclassified IBD, or patients with more active or severe disease is unclear.

Although impact and resistance-based exercise may be biologically plausible strategies for supporting bone health, the available evidence is insufficient to define an optimal exercise modality, frequency, intensity, progression, or loading strategy for improving aBMD in IBD. Exercise may still be recommended for its broader physical and psychosocial health benefits, but its specific role as a bone-targeted therapy in IBD remains unestablished.

### 5.2. Implications for Research

Future trials should aim to recruit more diverse populations in terms of sex, disease activity, and geographical setting. The predominance of female participants and the exclusion of individuals with moderate to severe disease activity limit the generalizability of current findings. Expanding the age range and including participants at different stages of disease duration may also provide important insights into the timing and effectiveness of exercise interventions for bone health.

To improve comparability across studies, future trials should prospectively register detailed primary and secondary outcomes, standardize and prespecify DXA sites (e.g., lumbar spine and total hip/femoral neck), and report absolute aBMD changes (g/cm^2^) alongside percentage changes. They should also report adherence, progression, exercise dose, adverse events, disease activity, corticosteroid exposure, nutritional status, and habitual physical activity. These details are essential to determine whether skeletal responses are driven by the prescribed mechanical loading stimulus, overall physical activity, or broader clinical and behavioral factors.

## Figures and Tables

**Table 1:** General description of the selected studies [19,20].

Study	Participant Characteristics		Comparator Condition	Objectives	Intervention	Outcomes
	Country	Population				
Jones et al., (2020) [19]	England	47 participants with a clinical diagnosis of CD; 32% males; age: exercise group, 46.1 ± 11.9 years; usual-care control group, 52.3 ± 13.6 years; total, 49.3 ± 13.0 years	Usual care	To assess the effect of combined impact and resistance training on aBMD and muscle function in adults with CD	6-month combined impact and resistance training program, involving three, 3 × 60 min/week and a gradual tapering of supervision to self-management compared to usual care	aBMD at the femoral neck (dual-energy X-ray absorptiometry) Maximum voluntary isometric and isokinetic strength of the elbow flexor and knee extensor muscles (isokinetic dynamometry) Handgrip strength (handgrip dynamometer) Lower-limb muscle endurance (30 s chair sit-to-stand) Upper-limb muscle endurance (30 s arm bicep curl test) Quality of life (Inflammatory Bowel Disease Quality of Life Questionnaire (IBDQ)) Health status (EuroQol 5-dimensions, 5-level questionnaire (EQ-5D-5L)) Fatigue (Inflammatory Bowel Disease Fatigue Scale (IBD-F)) Disease activity (Crohn’s Disease Activity Index (CDAI)) Bowel inflammation (faecal calprotectin) Blood markers of inflammation (C-reactive protein) Physical activity (Scottish Physical Activity Questionnaire (SPAQ))
Robinson et al., (1998) [20]	England	117 participants with CD confirmed by histological, endoscopic, radiological, and clinical criteria; 41% males; age: exercise group, 40.1 ± 12.6 years; no-intervention control group, 41.2 ± 14.1 years	No intervention	To investigate the effect of exercise on aBMD in patients with CD	12-month home-based low-impact exercise program of increasing intensity	aBMD (dual-energy X-ray absorptiometry) Physical activity (Allied Dunbar National Fitness Survey) Dietary intake (DIET Q)

Abbreviations: aBMD, areal bone mineral density; CD, Crohn’s disease.

**Table 2:** Summary of findings: Combined impact and resistance exercise compared with usual care, based on Jones et al. [19]. Participants: People with CD. Intervention: Combined impact and resistance training. Comparison: Usual care.

Outcomes	% Change (95% CI)	N° of Participants (Trials)	Certainty of the Evidence (GRADE)	Comments
aBMD: % change in aBMD at the lumbar spine (Follow-up: six months)	The % change in aBMD at the lumbar spine was higher in the exercise group than the control group, MD 3.5 (95% CI 2.3 to 4.6)	47 (1)	⊕⊝⊝⊝ very low ^a,b^	The evidence is very uncertain about the effect of exercise compared to usual care on the aBMD at the lumbar spine
aBMD: % change in aBMD at the femoral neck (Follow-up: six months)	The % change in aBMD at the hip was higher in the exercise group than the control group, MD 2.4 (95% CI 0.1 to 4.6)	47 (1)	⊕⊝⊝⊝ very low ^a,b^	The evidence is very uncertain about the effect of exercise compared to usual care on the aBMD at the femoral neck
aBMD: % change in aBMD at the greater trochanter (Follow-up: six months)	The % change in aBMD at the greater trochanter was higher in the exercise group than the control group, MD 1.9 (95% CI −2.8 to 6.6)	47 (1)	⊕⊝⊝⊝ very low ^a,b^	The evidence is very uncertain about the effect of exercise compared to usual care on the aBMD at the greater trochanter

Abbreviations: aBMD: areal bone mineral density; CD: Crohn’s disease; CI: confidence interval; MD: mean difference; ⊕⊝⊝⊝: Very low certainty evidence. GRADE Working Group grades of evidence. High certainty: We are very confident that the true effect lies close to that of the estimate of the effect. Moderate certainty: We are moderately confident in the effect estimate: The true effect is likely to be close to the estimate of the effect, but there is a possibility that it is substantially different. Low certainty: Our confidence in the effect estimate is limited: The true effect may be substantially different from the estimate of the effect. Very low certainty: We have very little confidence in the effect estimate: The true effect is likely to be substantially different from the estimate of effect. ^a^ Downgraded one level due to risk-of-bias concerns, mainly related to the absence of participant and facilitator masking, the lack of an attention-matched comparator, and some concerns about selective reporting or analytical detail. Lack of participant blinding was not considered sufficient by itself to judge DXA-derived aBMD as high risk of bias. ^b^ Downgraded two levels for imprecision because the estimate was based on one small RCT with 47 participants. The percentage aBMD values were calculated from adjusted between-group MDs in aBMD expressed in g/cm^2^, divided by the baseline aBMD value of the control group at the corresponding skeletal site and multiplied by 100. These values were included for interpretability only and did not alter the original statistical inference.

**Table 3:** Summary of findings: Low-impact exercise compared with no intervention, based on Robinson et al. [20]. Participants: People with CD. Intervention: Low-impact exercise program. Comparison: No intervention.

Outcomes	% Change (95% CI)	No. of Participants (Trials)	Certainty of the Evidence (GRADE)	Comments
aBMD: % change in aBMD at the lumbar spine (Follow-up:12 months)	The % change in aBMD at the lumbar spine was higher in the exercise group than the control group, MD 0.77 (95% CI −0.97 to 2.51)	117 (1)	⊕⊝⊝⊝ very low ^a,b^	The evidence is very uncertain about the effect of exercise compared to no intervention on the aBMD at the lumbar spine
aBMD: % change in aBMD at the femoral neck (Follow-up:12 months)	The % change in aBMD at the hip was higher in the exercise group than the control group, MD 0.5 (95% CI −1.39 to 2.39)	117 (1)	⊕⊝⊝⊝ very low ^a,b^	The evidence is very uncertain about the effect of exercise compared to no intervention on the aBMD at the hip
aBMD: % change in aBMD at the greater trochanter (Follow-up:12 months)	The % change in aBMD at the greater trochanter was higher in the exercise group than the control group, MD 0.86 (95% CI −1.41 to 4.1)	117 (1)	⊕⊝⊝⊝ very low ^a,b^	The evidence is very uncertain about the effect of exercise compared to no intervention on the aBMD at the greater trochanter

Abbreviations: aBMD: bone mineral density; CD: Crohn’s disease; CI: confidence interval; MD: mean difference; ⊕⊝⊝⊝: Very low certainty evidence. GRADE Working Group grades of evidence. High certainty: We are very confident that the true effect lies close to that of the estimate of the effect. Moderate certainty: We are moderately confident in the effect estimate: The true effect is likely to be close to the estimate of the effect, but there is a possibility that it is substantially different. Low certainty: Our confidence in the effect estimate is limited: The true effect may be substantially different from the estimate of the effect. Very low certainty: We have very little confidence in the effect estimate: The true effect is likely to be substantially different from the estimate of effect. ^a^ Downgraded two levels due to risk-of-bias concerns, including unclear allocation concealment, absence of participant blinding, lack of an attention-matched comparator, differential missing outcome data, and absence of a prospectively available protocol or trial registration. ^b^ Downgraded one level due to imprecision because the estimate was based on a single RCT and the confidence interval was compatible with both no effect and potential benefit. The percentage aBMD values were calculated from adjusted between-group mean differences in aBMD expressed in g/cm^2^, divided by the baseline aBMD value of the control group at the corresponding skeletal site and multiplied by 100. These values were included for interpretability only and did not alter the original statistical inference.

## Data Availability

No new data were created or analyzed in this study. Data sharing is not applicable to this article.

## Appendix A. Search Strategies for Each Database

- PUBMED

(“Osteoporosis” [Title/Abstract] OR “bone loss” [Title/Abstract] OR “bone mineral density” [Title/Abstract] OR “osteoporotic fractures” [Title/Abstract] OR “bone density” [Title/Abstract]) AND (“inflammatory bowel” [Title/Abstract] OR “ulcerative colitis” [Title/Abstract] OR “crohn’s” [Title/Abstract] OR “crohn” [Title/Abstract] OR “crohns” [Title/Abstract]) AND (“Walk” [Title/Abstract] OR “walking” [Title/Abstract] OR “run” [Title/Abstract] OR “running” [Title/Abstract] OR “swim” [Title/Abstract] OR “swimming” [Title/Abstract] OR “jog” [Title/Abstract] OR “jogging” [Title/Abstract] OR “biking” [Title/Abstract] OR “cycling” [Title/Abstract] OR “stretch” [Title/Abstract] OR “stretching” [Title/Abstract] OR “bike riding” [Title/Abstract] OR “resistance training” [Title/Abstract] OR “tai chi” [Title/Abstract] OR “weight lift” [Title/Abstract] OR “weight lifting” [Title/Abstract] OR “yoga” [Title/Abstract] OR “tennis” [Title/Abstract] OR “pilates” [Title/Abstract] OR “aerobic” [Title/Abstract] OR “fitness” [Title/Abstract] OR “exercise” [Title/Abstract])

- **SCOPUS**

Advanced query
(TITLE-ABS-KEY (osteoporosis OR bone-loss OR bone-mineral-density OR osteoporotic-fractures OR bone-density) AND TITLE-ABS-KEY (inflammatory-bowel OR ulcerative-colitis OR crohn’s OR crohn OR crohns) AND TITLE-ABS-KEY (walk OR walking OR run OR running OR swim OR swimming OR jog OR jogging OR biking OR cycling OR stretch OR stretching OR bike-riding OR resistance-training OR tai-chi OR weight-lift OR weight-lifting OR yoga OR tennis OR pilates OR aerobic OR fitness OR exercise))

- **CENTRAL**

#1 (osteoporosis): ti, ab, kw OR (“bone loss”): ti, ab, kw OR (“bone mineral density”): ti, ab, kw OR (“osteoporotic fractures”): ti, ab, kw AND (“bone density”): ti, ab, kw (Word variations have been searched)→21,301
#2 (“inflammatory bowel”): ti, ab, kw OR (“ulcerative colitis”): ti, ab, kw OR (crohn’s): ti, ab, kw OR (crohn): ti, ab, kw AND (crohns): ti, ab, kw (Word variations have been searched)→13,374
#3 (Walk): ti, ab, kw OR (walking): ti, ab, kw OR (run): ti, ab, kw OR (running): ti, ab, kw AND (swim): ti, ab, kw (Word variations have been searched)→71,993
#4 (swimming): ti, ab, kw OR (jog): ti, ab, kw OR (jogging): ti, ab, kw OR (biking): ti, ab, kw AND (cycling): ti, ab, kw (Word variations have been searched)→2903
#5 (stretch): ti, ab, kw OR (stretching): ti, ab, kw OR (“bike riding”): ti, ab, kw OR (“resistance training”): ti, ab, kw AND (“tai chi”): ti, ab, kw (Word variations have been searched)→11,800
#6 (“weight lift”): ti, ab, kw OR (“weight lifting”): ti, ab, kw OR (yoga): ti, ab, kw OR (tennis): ti, ab, kw AND (pilates): ti, ab, kw (Word variations have been searched)→7627
#7 (exercise): ti, ab, kw OR (aerobic): ti, ab, kw OR (fitness): ti, ab, kw (Word variations have been searched)→176,450
  #8 #3 OR #4 OR #5 OR #6 OR #7→224718
#9 #1 AND #2 AND #8→17

- **WOS**

TS = (Osteoporosis OR bone-loss OR bone-mineral-density OR osteoporotic-fractures OR bone-density) AND TS = (inflammatory-bowel OR ulcerative-colitis OR crohn’s OR crohn OR crohns) AND TS = (Walk OR walking OR run OR running OR swim OR swimming OR jog OR jogging OR biking OR cycling OR stretch OR stretching OR bike-riding OR resistance-training OR tai-chi OR weight-lift OR weight-lifting OR yoga OR tennis OR pilates OR aerobic OR fitness OR exercise)

## Appendix B. Risk of Bias of Included Studies and Judgement of Each Domain

**Robinson et al. (1998) [20]**		
**Bias Domain**	**Authors’ Judgement**	**Support for Judgement**
Bias arising from the randomization process	High risk	No method to conceal the allocation was described.
Bias due to deviations from intended interventions	Some concerns	Participants could not be blinded to exercise allocation, which is expected in exercise trials. For objective DXA-derived aBMD, this was not considered sufficient by itself to indicate high risk of bias. However, some concerns remain because the comparator was no intervention and co-intervention or behavioral differences between groups cannot be excluded.
Bias due to missing outcome data	High risk	In total 9% of the participants were lost to follow up. With a 2:1 ratio (intervention group: control group)
Bias in measurement of the outcome	Some concerns	aBMD was measured using DXA, an objective method. However, blinding of outcome assessors was not reported; therefore, some concerns remain.
Bias in selection of the reported result	Some concerns	No trial protocol or prospective registration was identified, so it was not possible to verify whether all prespecified outcomes and analyses were fully reported.

**Jones et al. (2020) [19]**		
**Bias Domain**	**Authors’ Judgement**	**Support for Judgement**
Bias arising from the randomization process	Low risk	Participants were randomly assigned using a computer-generated randomization schedule stratified by sex and baseline disease status. The randomization process was managed by an investigator who was not involved in recruitment, intervention delivery, or data collection, and allocation was disclosed after consent and baseline assessment.
Bias due to deviations from intended interventions	Some concerns	Participants and intervention facilitators were not masked to group allocation, which is expected in exercise trials. However, lack of participant blinding was not considered sufficient by itself to indicate high risk of bias for objective DXA-derived aBMD. Some concerns remain because the comparator was usual care rather than an attention-matched control, and performance-related differences between groups cannot be fully excluded
Bias due to missing outcome data	Low risk	In total 9% of the participants were lost to follow up. With a 1:3 ratio (intervention group: control group). However, the reasons were not linked with health status.
Bias in measurement of the outcome	Some concerns	For DXA-derived aBMD, risk of measurement bias was judged to be low because aBMD is an objective outcome and the trial was described as assessor-blind. For self-reported outcomes, such as physical activity, fatigue, and quality of life, some concerns remain because participants were not blinded to allocation.
Bias in selection of the reported result	Some concerns	The trial was prospectively registered, and aBMD outcomes were reported. However, the registry/protocol prespecified several primary and secondary outcomes, and some outcomes appear to have been reported with less analytical detail than others. Therefore, some concerns remain regarding possible selective reporting or selective analytical emphasis
